# ‘Molecular habituation’ as a potential mechanism of gradual homeostatic loss with age

**DOI:** 10.1016/j.mad.2017.11.010

**Published:** 2018-01

**Authors:** Alvaro Martinez Guimera, Ciaran M. Welsh, Carole J. Proctor, Anne McArdle, Daryl P. Shanley

**Affiliations:** a Institute for Cell and Molecular Biosciences (ICaMB), Ageing Research Laboratories, Campus for Ageing and Vitality, Newcastle University, Newcastle Upon Tyne, NE4 5PL,United Kingdom; b Institute of Cellular Medicine, Ageing Research Laboratories, Campus for Ageing and Vitality, Newcastle University, Newcastle Upon Tyne, NE4 5PL, United Kingdom; c Department of Musculoskeletal Biology, University of Liverpool (University, Not-for-profit), Institute of Ageing and Chronic Disease,William Duncan Building, 6 West Derby Street, Liverpool L7 8TX, United Kingdom; d MRC/Arthritis Research UK Centre for Musculoskeletal Ageing (CIMA), United Kingdom

**Keywords:** Oxidative stress, Redox signalling, Ageing, Information theory, Systems modelling

## Abstract

The ability of reactive oxygen species (ROS) to cause molecular damage has meant that chronic oxidative stress has been mostly studied from the point of view of being a source of toxicity to the cell. However, the known duality of ROS molecules as both damaging agents and cellular redox signals implies another perspective in the study of sustained oxidative stress. This is a perspective of studying oxidative stress as a constitutive signal within the cell. In this work, we adopt a theoretical perspective as an exploratory and explanatory approach to examine how chronic oxidative stress can interfere with signal processing by redox signalling pathways in the cell. We report that constitutive signals can give rise to a ‘molecular habituation’ effect that can prime for a gradual loss of biological function. This is because a constitutive signal in the environment has the potential to reduce the responsiveness of a signalling pathway through the prolonged activation of negative regulators. Additionally, we demonstrate how this phenomenon is likely to occur in different signalling pathways exposed to persistent signals and furthermore at different levels of biological organisation.

## Highlights

• Constitutive signals indicate homeostatic dysregulation but their effect on signal transduction remains largely unexplored.
• A theoretical approach is undertaken to examine how oxidative stress may affect redox signal transduction.
• Constitutive signals can result in a ‘molecular habituation’ effect that interferes with information transmission.
• The robustness of such a theoretical observation to the underlying methodology hints at the generality of this principle.

## 1 Introduction

Whilst reactive oxygen species (ROS) are known to be deleterious and unavoidable products of cellular metabolism, it is apparent that these molecules mediate essential signalling functions within cells (Winterbourn, 2015, Wang and Hai, 2016). Just a few examples of processes mediated by redox signalling include the modulation of insulin signalling (Besse-Patin and Estall, 2014), the stress response (Jiang et al., 2011), cell survival (Trachootham et al., 2008) and tissue regeneration (Sen and Roy, 2008). The elucidation of redox signalling pathways occurred in parallel to the accumulation of evidence that various tissues displayed markers of oxidative stress in various pathologies (Besse-Patin and Estall, 2014, Barbieri and Sestili, 2012, De Marchi et al., 2013, Kim et al., 2015, Sosa et al., 2013, Brioche and Lemoine-Morel, 2016, Lepetsos and Papavassiliou, 2016) and the ageing process (Sanz, 2016, Kirkwood and Kowald, 2012). The established double-edged nature of ROS raises questions as to how cells move from a state of controlled ROS production to a state of oxidative stress.

Oxidative stress is defined as a cellular state involving a mismatch between the abundance of oxidant molecules and the antioxidant capacity of the cell, favouring the former (Sies, 2015). The resulting elevation in the intracellular levels of oxidant can be transient or constitutive (Pickering et al., 2013). Transient (acute) oxidative stress is associated with redox signalling. Constitutive oxidative stress is associated with a prolonged state of elevated oxidant levels. Constitutive or chronic oxidative stress thus involves longer time-scales as is the case in chronic diseases, age-related diseases and the ageing process. Oxidative stress has drawn considerable attention due to the intrinsic reactivity of ROS (Winterbourn, 2015). This chemical property confers these molecules the capacity to cause molecular damage, consequently flagging them as potential causal agents of observed homeostatic disruptions in age and disease.

The perspective of studying oxidative stress as a constitutive signal within the cell has generated some insights: for example, how chronic oxidative stress can become a constant inhibitory signal in calcium signalling (Gorlach et al., 2015, Roedding et al., 2013) and T cell activation (Fulop et al., 2014). However, it remains unclear how redox signalling within cells may be affected by sustained oxidative stress. In other words, how will redox signalling pathways respond to an acute ROS signal on top of a constitutively elevated basal oxidant level in the cellular environment. This is of physiological significance, since redox signalling pathways have been shown to become dysfunctional in a variety of tissues in contexts where oxidative stress is also present in the cell (Sohal and Orr, 2012, McDonagh et al., 2014, Cobley et al., 2015, Vasilaki et al., 2006, Claflin et al., 2015, Jackson, 2016, Zhang et al., 2015, Done et al., 2016).

The problem becomes whether the constitutive presence of a signal in the environment affects a signalling pathway’s ability to transduce a subsequent acute pulse of the same signal. Whilst the reactivity of reactive oxygen species limits the resolution of current experimental methods (Woolley et al., 2013, Ribou, 2016), very few studies have looked at the effects of long-term exposure of cells to controlled oxidant levels (Covas et al., 2013, Tan et al., 2015, Millonig et al., 2012, Sobotta et al., 2013), with even fewer explicitly examining what effect this exposure would have on a subsequent acute redox signal fed through the system. Work published by Pickering et al. seems to indicate that a chronic exposure of cells to elevated oxidant levels can blunt redox-mediated adaptive responses (Pickering et al., 2013).

Testing all of the potential mechanisms via which chronic oxidative stress could affect physiological redox signalling would be a time-consuming endeavour. In this work, we adopt a theoretical perspective as an exploratory and explanatory approach to examine how chronic oxidative stress can interfere with signal processing by redox signalling pathways in the cell. We report that a constitutive signal in the environment has the ability to reduce the responsiveness of the signalling pathway through the prolonged activation of negative regulators. Additionally, we demonstrate how this phenomenon is likely to occur in different signalling pathways exposed to persistent signals and furthermore at different levels of biological organisation.

## 2 A rationale for a generic redox model

The major redox stress response pathways in the cell, i.e. NFκB, Nrf2, ASK1, HIF1 and HSF1, reveal conserved topological features. In all pathways cellular stress will interfere with an inhibitor-activator complex (Soga et al., 2012, Hoesel and Schmid, 2013, Tebay et al., 2015, Masoud and Li, 2015, Jiang et al., 2015): IκB − NFκB (Hoesel and Schmid, 2013); Keap1–Nrf2 (Tebay et al., 2015); Thioredoxin1–ASK1 (Soga et al., 2012); VHL − HIF1α (Masoud and Li, 2015) or HSP70/90–HSF1 (Jiang et al., 2015). Oxidant molecules will directly disrupt the inhibition of the activator molecule in the case of the Nrf2 and ASK1 responses, arguably the NFκB response (Oliveira-Marques et al., 2009, Morgan and Liu, 2011) and also the HIF1 response (Nanduri et al., 2015, Chandel et al., 2000). The case of HSF1 differs in that oxidative stress is likely to be sensed indirectly through the abundance of unfolded proteins or the activation of other pathways (Yoo et al., 2014, Swan and Sistonen, 2015). In any case, all pathways have been reported to be activated in response to an oxidant stimulus. It follows from this, that in all pathways the activator molecule must undergo a binding event with, or a modification by, a second molecule to be stabilised and perform a function. These are other ASK1 molecules in case of the ASK1 pathway or co-factors in the case of the Nrf2, NFκB, HIF1, HSF1 transcription factors. Additionally, a stabilising phosphorylation step has been reported for all molecules. Eventually the response must be terminated and these pathways must return to their original state: an activator being actively bound by an inhibitor to form an inactive complex. This requires the complex to be regenerated. Such regeneration occurs through the post-translational modification of the inhibitor, e.g. reduction, or through its *de novo* synthesis.

**Fig. 1:**
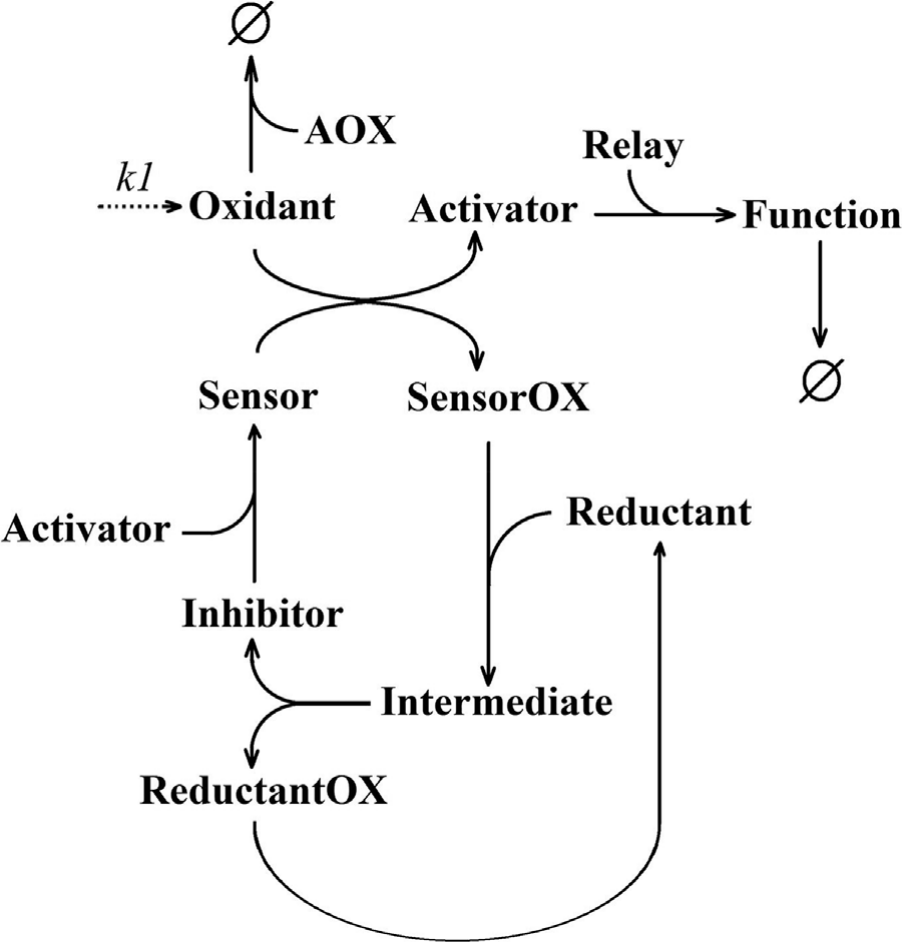
Network diagram of generic redox signalling Model 1. The steady-state level of the ‘Oxidant’ signal is modulated through the rate constant k1 which determines the flux of oxidant generation. An oxidation reaction will result in the ‘Activator’ escaping inhibition and performing a ‘Function’ after being stabilised by a binding event with a ‘Relay’ molecule. Meanwhile the inhibitor will undergo a two-step regeneration process before it is able to bind the ‘Activator’ into an inhibitory complex (Sensor). AOX=Antioxidant. OX suffix = oxidized. Slashed circle = degraded.

Based on these observations we defined a core generic redox model (Fig. 1), hereafter referred to as Model 1. Such core model consists of ‘Sensor’ molecules that can react with ‘Oxidant’ molecules which can additionally be scavenged by antioxidant molecules (AOX). Oxidation of ‘Sensor’ molecules to yield ‘SensorOX’ will cause the release of an ‘Activator’ molecule, which must first bind a ‘Relay’ molecule in order to be stabilised and be able to perform a function. The ‘Function’ molecule is used as a readout of the activity of the stabilised ‘Activator’ molecules. Oxidised sensor molecules (SensorOX) can be reduced through a two-step process involving the binding to a ‘Reductant’ molecule to form an ‘Intermediate’ complex which is then resolved. The reduced form of the sensor that is not bound to an ‘Activator’ molecule is termed as an ‘Inhibitor’ species since it can bind ‘Activator’ molecules to reform the ‘Sensor’ complex.

**Fig. 2:**
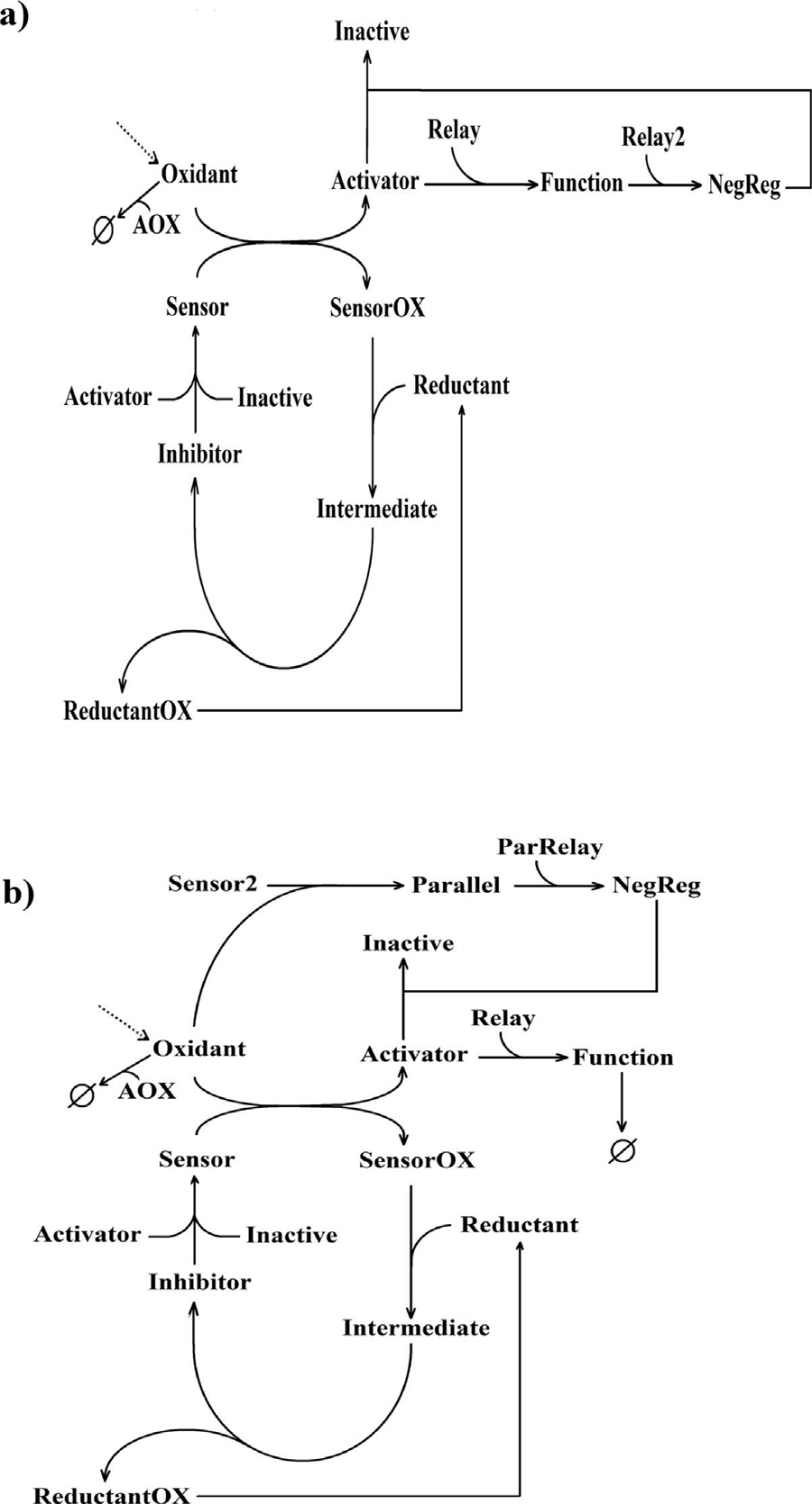
Network diagrams of generic redox signalling models incorporating a) a negative feedback loop (Model 2) or b) a negative feedforward loop (Model 3). AOX = Antioxidant. Slashed circle = degraded.

Model 1 ignores feedback mechanisms within the pathways. However, in the major redox stress response pathways outlined above, there are significant uncertainties with regards to the number of negative regulators in each pathway and their relative importance. Despite this, all the pathways have been reported to contain a negative feedback loop occurring through the activator-mediated transcription of inhibitor genes. The negative regulator that mediates the negative feedback loop is able to destabilise the activator in a first step and this results in the subsequent formation of the inhibitor-activator complex. This would correspond to the disruption of the transcriptional complexes and the subsequent nuclear export of NFκB, Nrf2, HSF1 and HIF1 transcription factors or the destabilisation of the ASK1 signalosome. Model 2 is an expansion of Model 1 that includes this element of negative feedback in a simplified time-scale (Fig. 2a). Within Model 2, a negative regulator entity ‘NegReg’ is introduced into the system downstream of the functional activity of the stabilised activator molecules. This is modelled as ‘NegReg’ being formed by the reaction between the functional readout molecule (Function) and a second relay molecule (Relay2). Negative feedback occurs by the ability of the ‘NegReg’ molecules to react with the ‘Activator’ molecules to render them ‘Inactive’.

In the case of the Nrf2 pathway, which is the major regulator of the antioxidant response in cells (Tebay et al., 2015), the main mechanism behind the response shutdown occurs through the delayed activation of GSK3β (Tebay et al., 2015, Cuadrado, 2015). Delayed activation does not depend on Nrf2 activity and occurs through a slower parallel branch to the activation of Nrf2 by oxidant molecules (Cuadrado, 2015, Kaspar et al., 2009). This is an example of a negative feedforward loop. In contrast to the negative feedback loop which follows an in-series structure, the parallel topology of a negative feedforward loop allows the negative regulator to be introduced independently from the activator molecule. Such pathway structure is incorporated into Model 3 as an expansion of Model 1 (Fig. 2b). Model 3 follows the same principle with regards to how the ‘NegReg’ species enters the system, with the key difference that it does not depend on the activity of stabilised ‘Activator’ molecules. In this model, ‘NegReg’ is introduced by a parallel signalling branch that is activated by the oxidation of a second sensor molecule (Sensor2) by ‘Oxidant’ molecules. The result of this oxidation is the formation of a species arbitrarily named ‘Parallel’ which will introduce the negative regulator after a time delay modelled by its reaction with a relay molecule (ParRelay). Note that for simplicity, the two sensor molecules in Model 3 are not modelled to compete for oxidant species. The equations and parameters of Models 1–3 can be found in Supplementary Tables 1–9. With Models 2 and 3 as generalised representations of redox signalling pathways (exemplified in Fig. S1), we set to investigate how a redox signal would feed into the system in the presence and absence of basally elevated oxidant levels.

## 3 Results

### 3.1 Pathway responsiveness to an acute redox signal is reduced when oxidant levels are constitutively elevated

**Fig. 3:**
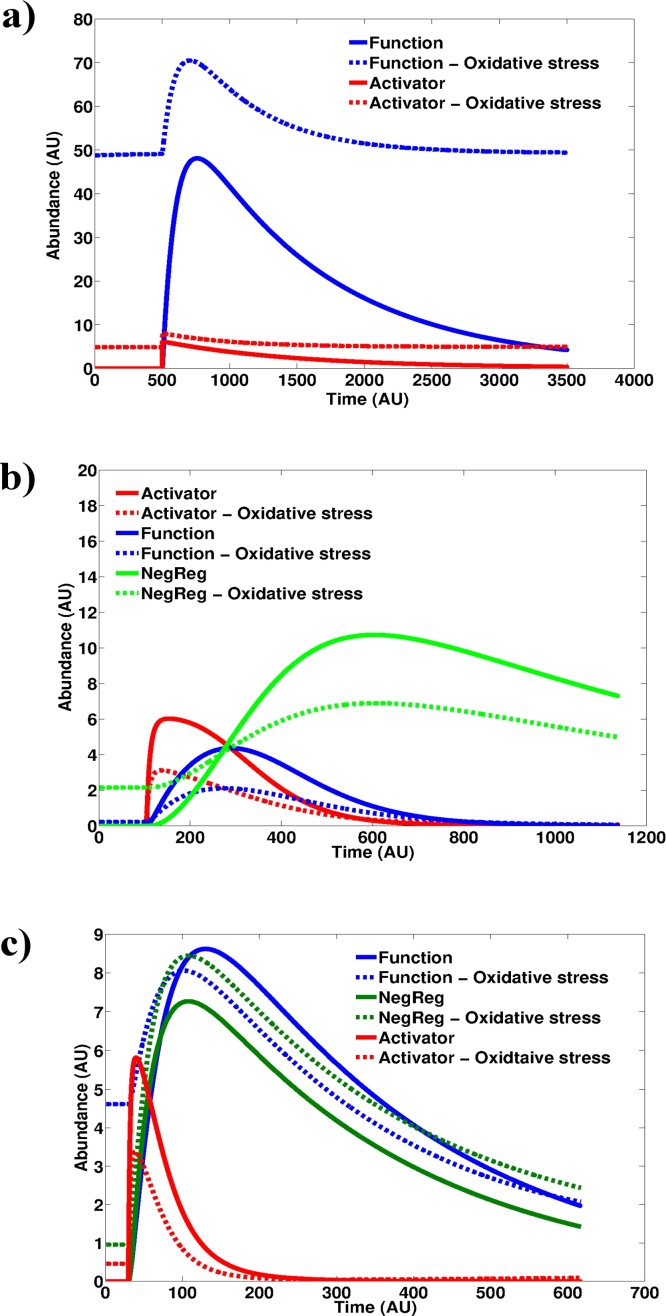
Oxidative stress reduces the response magnitude to an acute redox stimulus. Model 1 **(a)**, Model 2 **(b)** and Model 3 **(c)** were simulated at different values of basal oxidant levels. Absence of oxidative stress corresponds to a k1 value of 0 whilst oxidative stress corresponds to a k1 value of 1. Stimulus strength = 100.

The pathway activation profiles for both Model 2 and Model 3 displayed a lower response magnitude to the same redox signal when parameter *k1*, controlling the basal generation of oxidant, is increased (Fig. 3). A parameter scan reveals that with a step-wise increase in the rate of oxidant production, the response peaks in Models 2 and 3 trail off (Fig. S2a-d). This is indicative of the dampening effect being dependent on the relative values of *k1* and the stimulation strength, both of which are arbitrarily assigned in the models. The only exception to this observation is the *negative regulator* in Model 3, which displays an increased activation magnitude at higher values of *k1*. This is significant since the negative regulator in Model 3 is independent of the levels of the ‘Activator’ molecule suggesting the reduced sensitivity could stem from a reduced signal processing at the level of the *activator*. An increased steady-state level of oxidant is furthermore seen to result in a constitutively elevated level of the ‘Activator’, ‘Function’ and ‘NegReg’ molecules (Fig. 3). When we performed the same parameter scan on Model 1 however, we obtained typical saturation profiles (Fig. 3a) with the magnitude of pathway activation by the redox signal increasing with increasing *k1*.

A possible explanation for the observed loss in responsiveness is that the continuous flux of oxidant in the model is stabilising a new steady state which displays a reduced sensitivity at a whole-network scale (Dalle Pezze et al., 2014). To test for this we performed sensitivity analysis on all three models (Fig. S3). No obvious network-scale reduction in sensitivity is observed in any of the models. In fact, a gain in sensitivity to the rate of reduction of the oxidized inhibitor (*k6* in Model 2 and *k7* in Models 1&3) is seen in all models under the presence of a basally elevated level of oxidant. Upon a closer inspection of the sensitivity analysis data we observed that, under conditions of oxidative stress, ‘Activator’ and ‘Function’ molecules displayed the highest sensitivity to parameters directly involving the negative regulation process. These are parameters *k10/k12* in Model 2 and *k10/k11/k12* in Model 3.

### 3.2 The constitutive elevation of the basal levels of negative regulators by oxidative stress drives the loss of pathway responsiveness

**Fig. 4:**
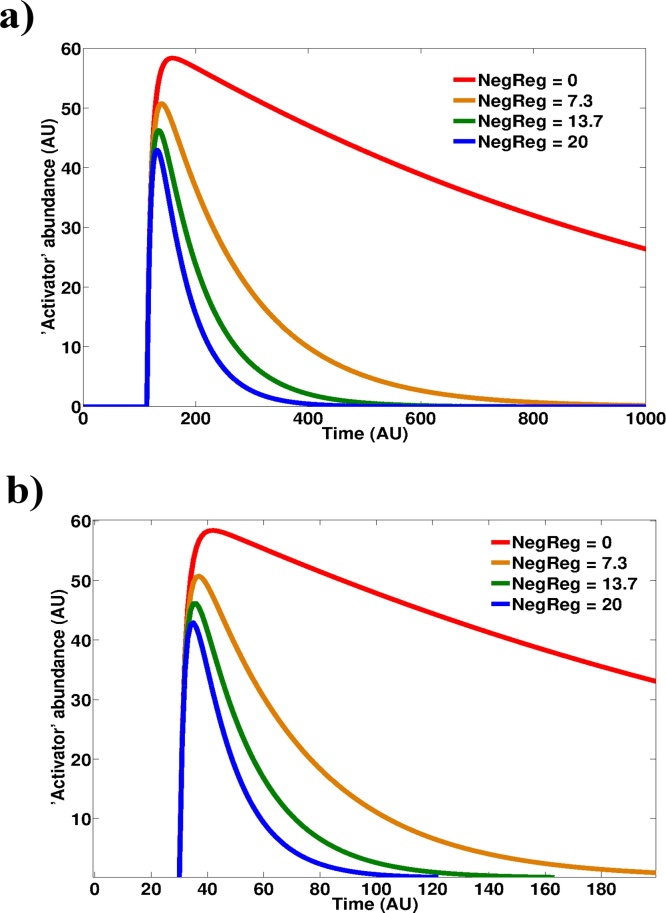
An increase in the basal levels of negative regulators reproduces the response dampening observed under conditions of oxidative stress. Simulations of pathway activation in Model 2 **(a)** and Model 3 **(b)** by an acute stimulus were run at different steady state levels of negative regulator (NegReg) molecules. Plots are shown for ‘Activator’ species. Rate constant k1 was set to zero and ‘NegReg’ abundance clamped at 4 different abundances. Stimulus strength = 100.

The main difference between Model 1 and Models 2 and 3 is that the latter two contain a negative regulator. It thus seems apparent that the basally elevated level of negative regulator stabilised by a sustained oxidant flux could provide a constant source of dampening of the redox signal. Should this be the case then the same response-blunting observation should be observed if, instead of altering the steady state levels of oxidant, the steady state levels of the negative regulator were clamped to fixed values and the system was stimulated with the same redox signal. When we performed this experiment *in silico* we observed a reduced activation magnitude with increasing levels of negative regulator (Fig. 4). We additionally checked that the blunting behaviour observed with increasing levels of oxidant production was robust to parameters controlling negative regulation strength and additionally independent on how this negative regulation was modelled, i.e. mass-action kinetics vs competitive inhibition kinetics (data not shown).

The behaviours displayed by our kinetic models are determined by the underlying ordinary differential equations. Thus, there is an implicit assumption that our adopted modelling framework is a good representation of the underlying biological process, and we can model cellular signalling pathways as molecular fluxes distributed across reaction branches. In order to be confident that our finding was robust to the modelling framework employed to model the signalling pathways, we simulated Models 2 and 3 as particles undergoing Brownian motion within an enclosed container in a 3D cellular automaton model. The aim being to confirm our findings in a modelling framework that does not rely on solving differential equations but where simulated profiles arise from first principles, that is, the Brownian movement of particles in three dimensional space. These simulations showed the same behaviour involving a reduced pathway activation alongside the presence of elevated negative regulator levels under conditions of oxidative stress (Fig. S4).

### 3.3 The observed behaviour is robust to variations in model topology

**Fig. 5:**
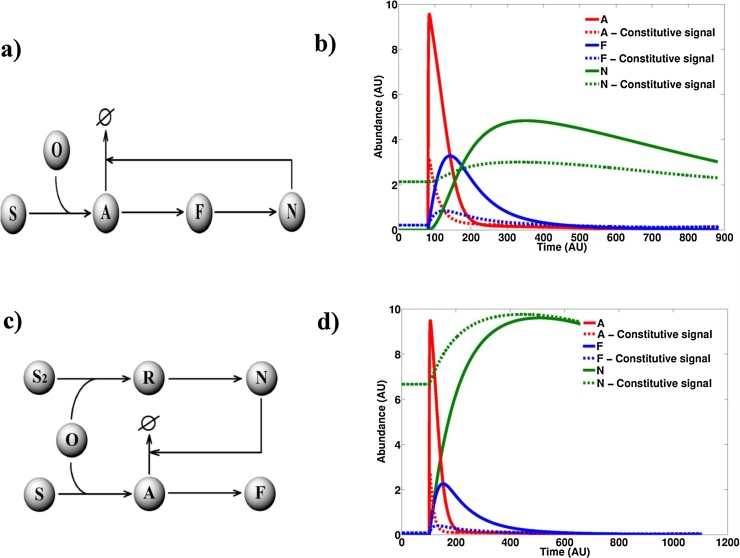
Generic circuits of negative regulation display a reduced response to an acute stimulus under the presence of a constitutive signal. a) Network diagram of Model 4. b) Deterministic simulation of Model 4 under k1 value of 0 (continuous line) and 0.02 (dashed line). c) Network diagram of Model 5. d) Deterministic simulation of Model 5 under k1 value of 0 (continuous line) and 0.02 (dashed line). k1 is the rate constant for O generation. Slashed circle = degraded. Stimulus strength = 100.

When formalising computational models of cellular signalling pathways there is always a degree of uncertainty regarding the representativeness of the underlying network structure. In order to check the robustness of the blunting behaviour to the model structures we further abstracted Models 2 and 3 to Models 4 and 5 respectively (Fig. 5a&c). Such a further abstraction could provide clues as to whether the reported effect of constitutive signals could affect generic circuits of negative regulation that are not necessarily part of redox signalling pathways. These simplified representations of a negative feedback loop and a negative feedforwards loop still displayed the same loss in responsiveness to an acute signal when the same signal was constitutively present in the environment (Fig. 5). This is, again, attributable to a steady state with basally elevated levels of the negative regulator *N*.

**Fig. 6:**
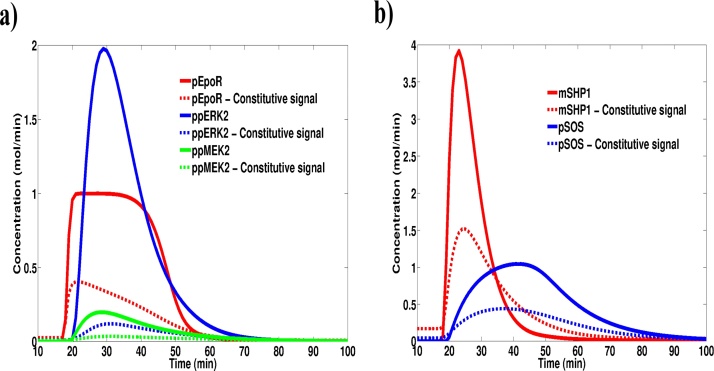
The dampening effect of constitutive signals is not unique to redox signalling systems. The ERK signalling model published by Shilling et al. (48) was simulated in the presence and absence of a constitutive Epo signal. a) Activator molecules in the pathway. b) Inhibitor molecules in the pathway. The constitutive signal corresponds to a rate constant of Epo generation of 0.1. The modification of the original model only involved the addition of two reactions; i) Epo generation following zero-order kinetics and ii) Epo degradation occurring through a first order mass action reaction with a rate constant of 0.1.

Sensitivity analysis of Models 4 and 5 showed a similar shift in model sensitivities as Models 2 and 3 (Fig. S5). Additionally, it is of interest to look for this blunting behaviour in models that are more complex, calibrated with experimental data and from an entirely different pathway. We performed a minor alteration to the extracellular-signal regulated kinase (ERK) signalling model published by Schilling et al. (Schilling et al., 2009) involving the addition of a reaction for Erythropoietin (Epo) synthesis and a reaction for Epo degradation. This minor modification constituted an elevated steady state level of this molecule on top of which the signalling event within the model would occur. Once again, we observed a reduced activation magnitude for this signalling pathway resulting from consistently elevated levels of the negative regulator molecules (Fig. 6).

### 3.4 The constitutive presence of a signal will cause a reduced information flow through the signalling pathway

**Fig. 7:**
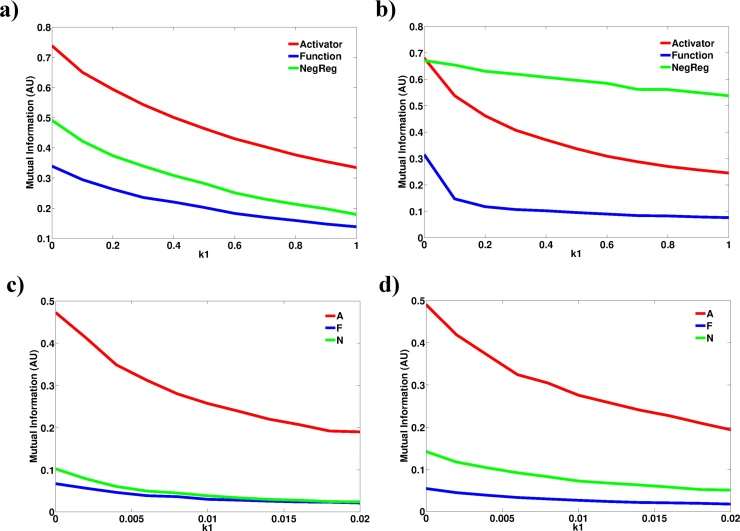
Constitutive signals reduce information flow through signalling pathways. Mutual information between model species and the input signal was quantified in Model 2 **(a)**, Model 3 **(b)**, Model 4 **(c)** and Model 5 **(d)** at different k1 values. ‘A’, ‘F’ and ‘N’ refer to molecular species in Models 4 and 5. Mutual information was calculated from the molecule distributions at the response peak derived from 1000 stochastic simulations. Stimulus strength = 100.

What is the functional consequence of the observed reduction in pathway responsiveness? This is a non-trivial question since although the activation magnitude may be reduced it is unclear if the resulting magnitude would still be large enough to trigger a response in the cell. Conceptually, a cell will trigger a response to a stimulus once the most downstream element of the signalling pathway undergoes a sufficient change from its basal abundance. The functional response thus depends on how well the downstream signalling molecule (Y) maps to the changes that occur in the upstream signalling molecule (X). How much information the state of Y provides about the state of X can be quantified through the use of mutual of information (Shannon, 2001). Mutual information analysis has been extensively used to quantify information transmission by biological signalling pathways (Waltermann and Klipp, 2011, Rhee et al., 2012, McMahon et al., 2015, Uda and Kuroda, 2016, Bowsher and Swain, 2014). Analysis of the mutual information between the signal molecule and the downstream signalling molecules in all models reveals that an increasing basal level of the signal will reduce the information flow through the signalling pathways (Fig. 7). The functional consequence of this reduced information flow can be appreciated in the simulated dose-response curves (Fig. S6), where stimulation magnitudes can be less clearly mapped to abundance values for molecule *F* in the presence of a constitutive signal. Thus, the presence of a constitutive signal can be a source of uncertainty to the cell regarding the actual level of the signal in the environment and consequently whether a response should be mounted or not. Such signalling dysregulation at the molecular level could impact at the tissue scale as populations of cells that fail to respond appropriately to a physiological signal.

### 3.5 Reduced responsiveness can occur across levels of biological organisation

**Fig. 8:**
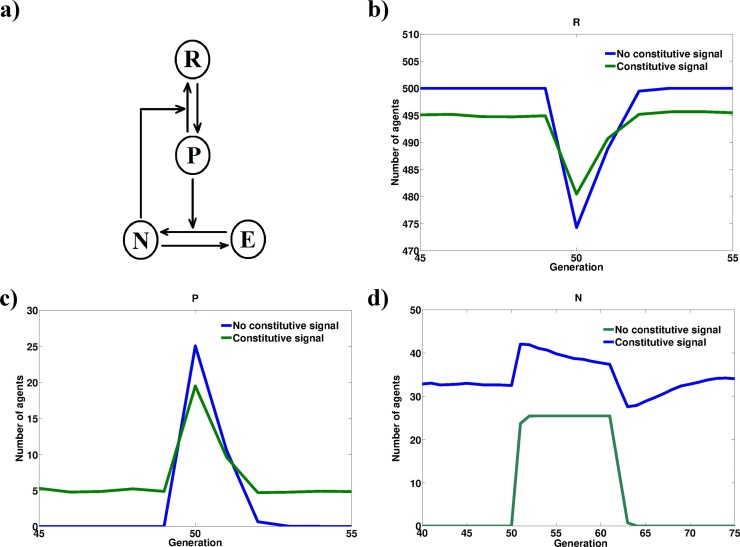
The response dampening by persistent inputs could occur at higher levels of biological organisation. Interacting agents were simulated in a three dimensional cellular automaton grid. a) Interaction map between the modelled agents. b) Time course for R agents. c) Time course for P agents. d) Time course for N agents. Constitutive signal corresponds to a basal induction probability P_ind_ value of 0.01. Stimulation is modelled as an increase in P_ind_ to 0.05 for a single generation. The absence of a constitutive signal corresponds to a basal P_ind_ of 0. Simulation run 1000 times at G_i_ = 10, n_i_ = 1, P_rec_ = 1, P_res_ = 0.9 and average agent populations plotted.

The level of abstraction in Models 4 and 5 suggest that the observed reduction in system response as a result of constitutively present negative regulators may not be restricted to molecular systems involved in intracellular signalling. Indeed, the mathematical formalisms behind Models 4 and 5 are sufficiently abstract to represent a system of interacting cellular or animal populations. To test this, we developed a 3D agent-based model through a cellular automaton framework. In this model agents begin at a resting state (*R*) and can transition to a perturbed state (*P*) which leads to the recruitment of agents (*N*) that induce a return to the resting state and become non-existent (*E*) from the simulation after a defined number of generations (Fig. 8a). All state transitions are probabilistic except for the lifetime of the *N* state. A transient change in the probability of transition from state *R* to state *P* causes a transient shift in the agent populations which is smaller in magnitude when the basal transition probability is higher (Fig. 8b–d). This is observed alongside an increased steady state population of *N* agents. Not only do these results re-inforce the independency of the blunting phenomenon to the underlying computational framework and interaction topology, but it also suggests that it could be observed at higher levels of biological organisation such as inter-cellular signalling.

## 4 Discussion

The ageing process and indeed many other chronic and acute pathological conditions are associated with a loss of homeostasis that often manifests as a broad range of signalling dysfunctionalities. Within the ageing process, it is still unclear how the loss of homeostasis may arise. However, it is known that oxidative stress is a common consequence of such loss of homeostasis, with the potential to cause molecular damage and drive the ageing process. In this work we show how oxidative stress can be viewed as a constitutive signal in the cellular environment that ultimately hinders the cell’s ability to mount a response to a physiological redox signal. Our observations seem to transcend the case of oxidative stress alone and be applicable to other pathways and levels of biological organisation. For instance, the same loss in responsiveness would be expected to be seen in the case of chronic inflammation, where inflammatory signals are constantly present in the cellular environment. In this context, the cells within a tissue would mount physiological responses which would be likely to be of insufficient strength, at an altered time and of an altered duration. Such state of quasi-functionality would be expected to lead to a gradual accumulation of damage and functional decline.

The basis behind the reduced responsiveness in biological systems exposed to a constant environmental signal is due to an increased basal level of negative regulator entities. It has been shown that cells are able to modulate pathway responsiveness through the abundance of negative regulator molecules (Toyoshima et al., 2012). Furthermore, the sustained presence of negative regulators has been associated with physiological habituation responses (Lee et al., 2013, Ramaswami, 2014, Grissom and Bhatnagar, 2009, Herman, 2013). Additional evidence comes from work in *Candida albicans* where the sustained activation of SAPKs stabilise a new steady state of sub-maximal Hog1 activation through an increase in the basal levels of the PTP negative regulators (Day et al., 2017). Dues et al. (Dues et al., 2016) systematically tested the ability of *C. elegans* to activate stress responses to a variety of stresses. The authors report a loss in responsiveness of all the stress responses in *C. elegans*, albeit not accompanied by an increase in their basal activation, with age (Dues et al., 2016). It is thus a possibility that the loss in responsiveness could serve an adaptive function which results in adverse effects when the constitutive signal persists for too long.

Such a possibility seems in line with the concept of hormesis, where a constant exposure to a low-level stress reduces the susceptibility to further perturbation (Yun and Finkel, 2014). A case has been made that the phenotypic culprit of biological ageing arises from the persistent presence of antagonistic hallmarks (Lopez-Otin et al., 2013). Antagonistic hallmarks, like cellular senescence, are a response to damage that initially serve a protective function but then become deleterious when constitutively present (Lopez-Otin et al., 2013). The reported effect of constitutive signals on both pathway responsiveness and information transmission appears to be in accordance with this notion. Supporting the idea of a short term benefit of pathway dampening by constitutive signals is the observation that the extended presence of negative regulators can be used as a refractory mechanism in cellular signalling (Adamson et al., 2016, Vizan et al., 2013).

Negative feedback loops have been shown to be able to lock biological systems in stable intermediate states under conditions of weak activation (Rahi Sahand et al., 2016). Their importance in shaping cellular signalling responses could lie behind the observation that negative regulators can correlate better with lifespan than the activator molecules they regulate (Lewis et al., 2015). In accordance with this is the observation within our simulations that the negative regulator provides a higher mutual information about the signal molecule than the ‘Function’ or ‘F’ molecules in Models 2–5. In the case of ageing and chronic disease, it thus seems relevant that the constitutively elevated levels of signalling molecules could be actively maintaining a state of dysfunctional signalling. It is worth noting that constitutive signals could potentially stabilise a new less responsive steady state through the sustained activation of positive feedback loops or other network sub-structures that display bistability (Shiraishi et al., 2010). Constitutive signals could in such way draw the system closer to a critical transition (Scheffer et al., 2009). In fact it could be speculated that the stochastic activation of these network sub-structures with the potential for bistability could be the source of the constitutive signal in the first place (Faucon et al., 2014). It would be an interesting possibility that these structures could provide an architectural weak-point within biological networks.

Whilst it is not always trivial to predict the consequences of a fractional loss in the responsiveness of a given biological system, an interesting perspective has been put forward by Dalle Pezze et al. (Dalle Pezze et al., 2014) who demonstrate how a reduction in network sensitivity can result in a loss of functionality since *‘the global decrease in sensitivity upon kinetic rate constants indicated that the semantics of these model parameters, e.g. promoter or inhibitor, became more uncertain. As a consequence, this uncertainty increased system noise and decreased network robustness, which, in the context of a cell, translated into weak signalling regulations and therefore poor intervention effectiveness’.*

A finding of particular interest is the ability of constitutive signals to reduce information flow through signalling pathways. Meaning that downstream effectors in signalling pathways will be less able to accurately reflect changes in the upstream signalling molecules. This translates into an increased heterogeneity in the cellular responses to physiological signals and therefore a greater proportion of cells that fail to respond appropriately. This increase in heterogeneity has been associated with a reduced system-level responsiveness and reduced effectiveness of interventions in senescent cells (Dalle Pezze et al., 2014). Interestingly, we found that the loss of information transmission through Models 2–5 with increasing basal levels of the constitutive signal closely resembles the loss in synaptic transmission efficacy of the snail *Helix aspersa* during habituation (Prescott and Chase, 1999). However, it should be noted that the models analysed do not display ultrasensitive behaviour. The mapping of an input signal to the on/off state of a downstream effector instead of a range of abundance values could mean the cell is more robust to losses in information transmission.

Importantly, a loss in information flow through signalling pathways means that cells will likely respond to signals with an altered magnitude, duration and/or timing. This state of quasi-functionality will likely be sufficient to maintain function but sub-optimal responses will also be likely to prime the cell for further damage and dysregulation and potentially drive a gradual loss of function typical of ageing processes. Such a state of quasi-functionality can result in subtle cellular- and tissue- level changes in the short term that only develop into an obvious loss of homeostasis in the long term. This is a new perspective on the propagation of signalling dysfunctionality across biological networks during the ageing process.

Other age-related changes that may affect the cellular regulatory machinery, such as altered expression of key sensor molecules or altered intermolecular binding affinities, are also likely to reduce information flow through signalling pathways. This suggests that information theory is an intuitive framework from which to understand loss of function and regulation during the process of biological ageing. An information-theoretic perspective has so far been overlooked and is likely to apply to be applicable to a wide range of biological systems that display age-related alterations. We believe such framework can mechanistically bridge the gap between the concept of stochastic damage in an organism lifetime and the observed gradual homeostatic decline with age.

It is important to note, especially in the case of redox signalling, that the loss of pathway responsiveness through the constitutive presence of negative regulators is likely to be acting in concert with other phenomena that would also affect pathway responsiveness. Examples could include the reduced expression of key sensor proteins, altered binding affinities that can result from genetic mutations or a reduced gradient across a membrane. In the case of oxidative stress, a redox signal generated in the mitochondria may encounter a reduced gradient due to a more oxidized cytosolic environment and so the same ROS pulse would experience a lower flux through the mitochondrial membrane. A constitutive low-level cysteine hyperoxidation could also be expected to blunt the responsiveness of redox signalling pathways to an acute redox stimulus.

A criticism that may arise from kinetic computational models is that they are often too simplistic and indeed appear “insulated” from the myriad of interactions that occur within the dense biological interaction networks. Questions also arise regarding how much of the parameter space supports the blunting behaviour within a given model topology. This criticism is the same as that for the analysis of network motifs (Ingram et al., 2006). We argue that, as is the case with network motifs, some observed biological dysregulation will be able to be mapped to this phenomenon in certain contexts and others maybe not so. It is also important to consider that should a biological system display perfect adaptation or dynamic compensation (Karin et al., 2016), it would be expected that constitutive signals might not be a source of dysfunctionality unless they interfered with the ability of the system to display such behaviours. For example, if the constitutive signal feeds into the system through crosstalk.

The loss of biological homeostasis in ageing and disease is expected to manifest as a constitutive elevation in signalling molecules, even if such a process arises from the constitutive downregulation of other molecules. This work is indicative of how a local loss in homeostasis in a cellular sub-system can spread through large portions of biological networks, potentially promoting a systemic dysregulation. Overall, it is informative to bear in mind that constitutive signals can lock signalling pathways into less responsive states. This is relevant for some diseases such as cancer where recognisable constitutive signals drive or stabilise a phenotype. It is also applicable to the ageing process, so often accompanied with the appearance of chronically elevated signals such as calcium (Decuypere et al., 2011), hormone imbalances (Maggio et al., 2013), inflammatory factors (Franceschi and Campisi, 2014) and oxidative stress.

In summary, this work reports a process of ‘molecular habituation’ within biological signalling pathways as a potential mechanism of signalling dysregulation during biological ageing. Such a phenomenon should be recognised by the seemingly paradoxical observation of a chronic activation of a signalling pathway occurring alongside an elevation in the levels of at least some of the negative regulators of such pathway.

## 5 Materials and methods

### 5.1 Signalling pathway simulation and analysis

The signalling pathway computational models are all constituted entirely by reactions following mass action kinetics unless specified otherwise. All deterministic simulations were run in COPASI (Hoops et al., 2006) with the LSODA parameters for relative and absolute tolerance set to 1e-06 and 1e-12 respectively and maximum internal steps set to 10000. Parameter scans were performed in COPASI. All stochastic simulations were run in Matlab’s Simbiology toolbox using the SSA solver. Note that in all types of simulations, an acute stimulus was simulated as an Event at a time point following the previous equilibration of the system to a steady state. All models have been deposited in the BioModels database (Chelliah et al., 2015) under the following identifiers; Model 1 − MODEL1710260000, Model 2 − MODEL1710260001, Model 3 − MODEL1710260002, Model 4 − MODEL1710260003, Model 5 − MODEL1710260004.

### 5.2 Molecular dynamics (MD)

Cellular automaton models can be used to simulate signalling pathways (Wurthner et al., 2000) and reaction kinetics (Schnell and Turner, 2004). A simple 3D Lattice Gas Cellular Automaton (LGCA) (Hatzikirou and Deutsch, 2010) simulator was developed in Matlab. The lattice-size is derived from a user-defined percentage occupancy of all of the specified initial molecule abundances. Individual cells in the lattice are treated as equal-sized molecules or empty space. User-defined molecular abundances are uniformly seeded at random in the lattice space. Molecular motion is simulated by a single-unit step-size random walk in a Moore neighbourhood. Upon encounter in space molecules react with a user-defined probability provided they have been defined as substrates. The outcome of a reaction is the replacement of the reactant molecules with product molecules and/or empty space. Thus, substrates that are not utilized in reactions (modifiers) are defined as both reactants and products of the reaction.

*De novo* synthesis reactions are simulated by the randomised uniform seeding of a user-defined number of molecules into the grid, with a user-defined probability, every generation. A signal is simulated as an assignment rule (Event) in the simulation where a user-defined molecular quantity is randomly distributed across the lattice at a specified generation. Encounter of non-substrate molecules or movement towards a cell outside of the grid will result in an 180° perfectly elastic collision.

### 5.3 Mutual information

A Matlab script was devised to calculate the mutual information (Shannon, 2001) between variables within the different computational models. The mutual information between two discrete random variables *X* and *Y* is defined as:

(1) $$I\left(X;Y\right)=\underset{x\in X}{\sum }\underset{y\in Y}{\sum }p\left(x,y\right)\cdot log\frac{p\left(x,y\right)}{p\left(x\right)\cdot p\left(y\right)}$$

However, in practice, the joint distribution p(x,y) is not a direct observable. Thus we can derive:

$$p\left(y|x\right)=\frac{p\left(x\cap^{\text{⁣}}y\right)}{p\left(x\right)}$$

$$p\left(x,y\right)=p\left(x\cap^{\text{⁣}}y\right)=p\left(x\right)\cdot p\left(y\text{|}x\right)$$

Substitute into Eq. (1) so then mutual information becomes:

(2) $$I\left(X;Y\right)=\sum_{x\in X}\sum_{y\in Y}p\left(x\right)\cdot p(y|x)\cdot log\frac{p(y|x)}{p\left(y\right)}$$

Consider *X* to be the upstream signalling molecule and *Y* to be the downstream protein effector. The calculation of the theoretical mutual information requires a simulated dose-response where, for each individual value *x* of the signal, the computational model must be stochastically simulated enough times to derive a consistent frequency tabulation of all possible abundance values of *Y* at a single time point or time window. Hence, *p(y|x)* is derived for each value of *X* and *p(y)* can be obtained from the marginal probabilities across all values of *X*. *p(x)* can be assumed to be a uniform distribution (Uda and Kuroda, 2016).

### 5.4 Agent model

A 3D cellular automaton model was constructed to simulate a regular lattice 50% occupied by agents seeded randomly across the grid. All agents begin in a resting state (*R*) and can transition to a series of other states throughout the simulation, namely, to a perturbed state (*P*), to a negative regulator state (*N*) or to a state of non-existence (*E*). At each generation, each randomly-selected agent can undergo the following rules:

i) If state is (*R*) then transition to (*P*) with probability *P_ind_*
ii) If state is (*P*) then *n_i_* neighbouring (*E*) agents transition to (*N*) with probability *P_rec_*
iii) If state is (*N*) then neighbouring (*P*) agents transition to (*R*) with probability *P_res_*
iv) If state is (*N*) and state has existed for *G_i_* generations then (*N*) transitions to (*E*)

Note that neighbouring agents are defined as those within the Moore neighbourhood of the selected agent. *n_i_* encodes the strength of negative regulation. Rule *G_i_* represents the duration of the presence of a negative regulatory state.

### 5.5 Sensitivity analysis

Local sensitivity analysis was carried out in COPASI. This method systematically and sequentially alters parameter values by a user-defined magnitude (as a percentage of the parameter value) and examines how simulation output is changed by such alteration (Kent et al., 2013). Normalised sensitivities were plotted in Matlab (MathWorks Inc., Natick, MA, 2016). Sensitivity analysis was performed at steady state and configured with parameter values of 0.001 and 1e-12 for the delta factor and the delta minimum respectively. Sensitivity analysis was performed on all kinetic rate constant parameters.

## Author summary

Ageing and disease can be understood as a loss of biological homeostasis. This commonly manifests as the constitutive elevation of biological signals. However, how such chronic elevation in the basal level of biological signals may affect regulatory pathways remains largely unexplored.

In this work we undertake a theoretical approach to explore how constitutive signals may affect the ability of biological systems to mount appropriate responses. We take oxidative stress feeding into redox signalling pathways as a working example.

We report that constitutive signals can reduce the responsiveness of biological signalling pathways by increasing the basal levels of negative regulator entities. The robustness of such a theoretical observation to the underlying methodology hints at the generality of this principle.
