# Supplementary material for: ‘Molecular habituation’ as a potential mechanism of gradual homeostatic loss with age
Source: Mech Ageing Dev. 2018 Jan;169:53–62. doi: 10.1016/j.mad.2017.11.010 (PMC5846846; doi:10.1016/j.mad.2017.11.010)
Supplement: Supplementary file 1 [file mmc1.docx]

***Supplementary Material***

**‘Molecular habituation’ as a potential mechanism of gradual homeostatic loss with age**

**Supplementary Figures**

**
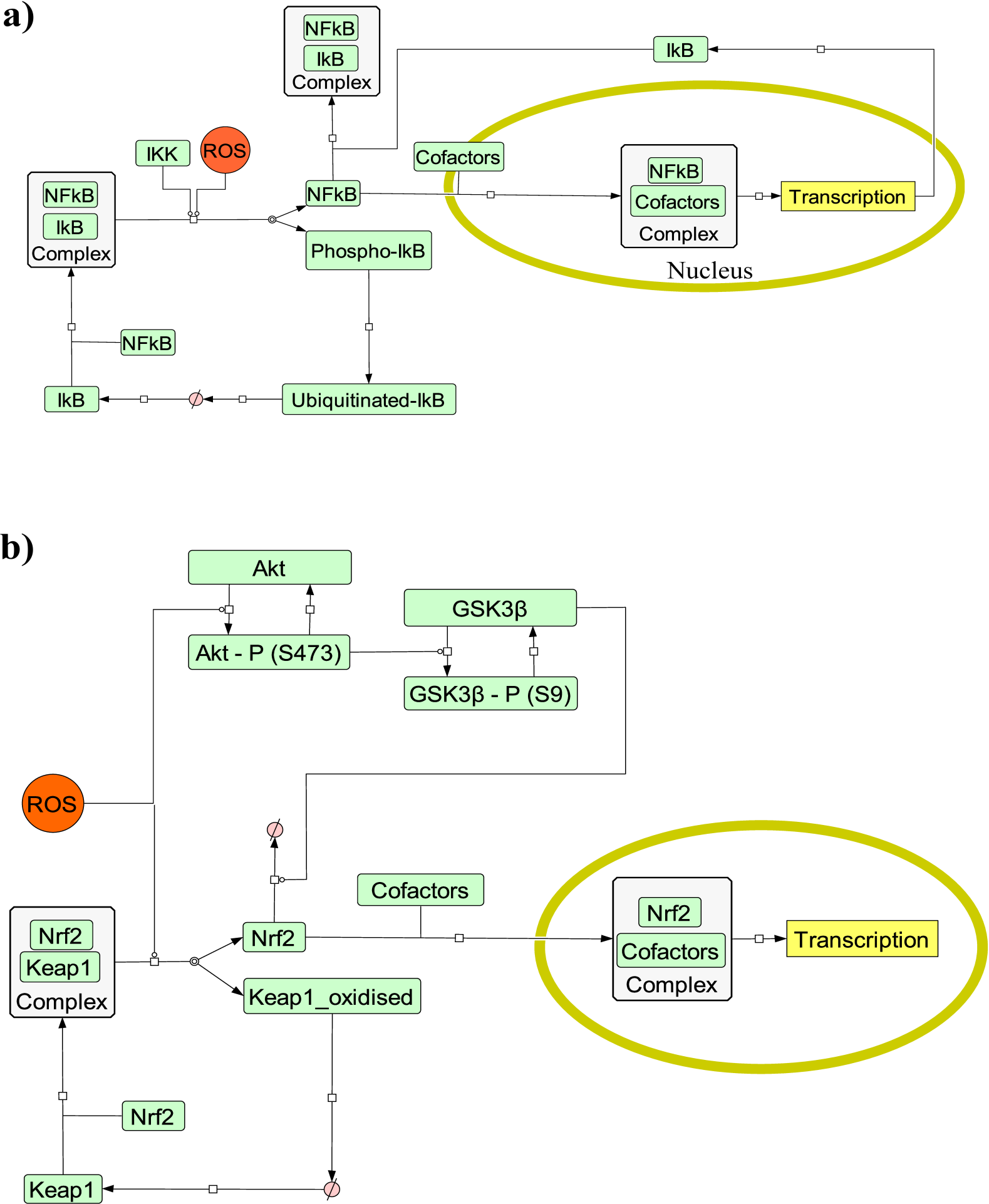
**

**Figure S1.** (Previous page) Simplified network diagrams of the NFκB signalling pathway exemplifying Model 2 **(a)** and the Nrf2 signalling pathway exemplifying Model 3 **(b)**. Dashed circle represents a state of non-existence so that arrows towards it indicate degradation reactions and arrows out of it represent synthesis reactions.

***
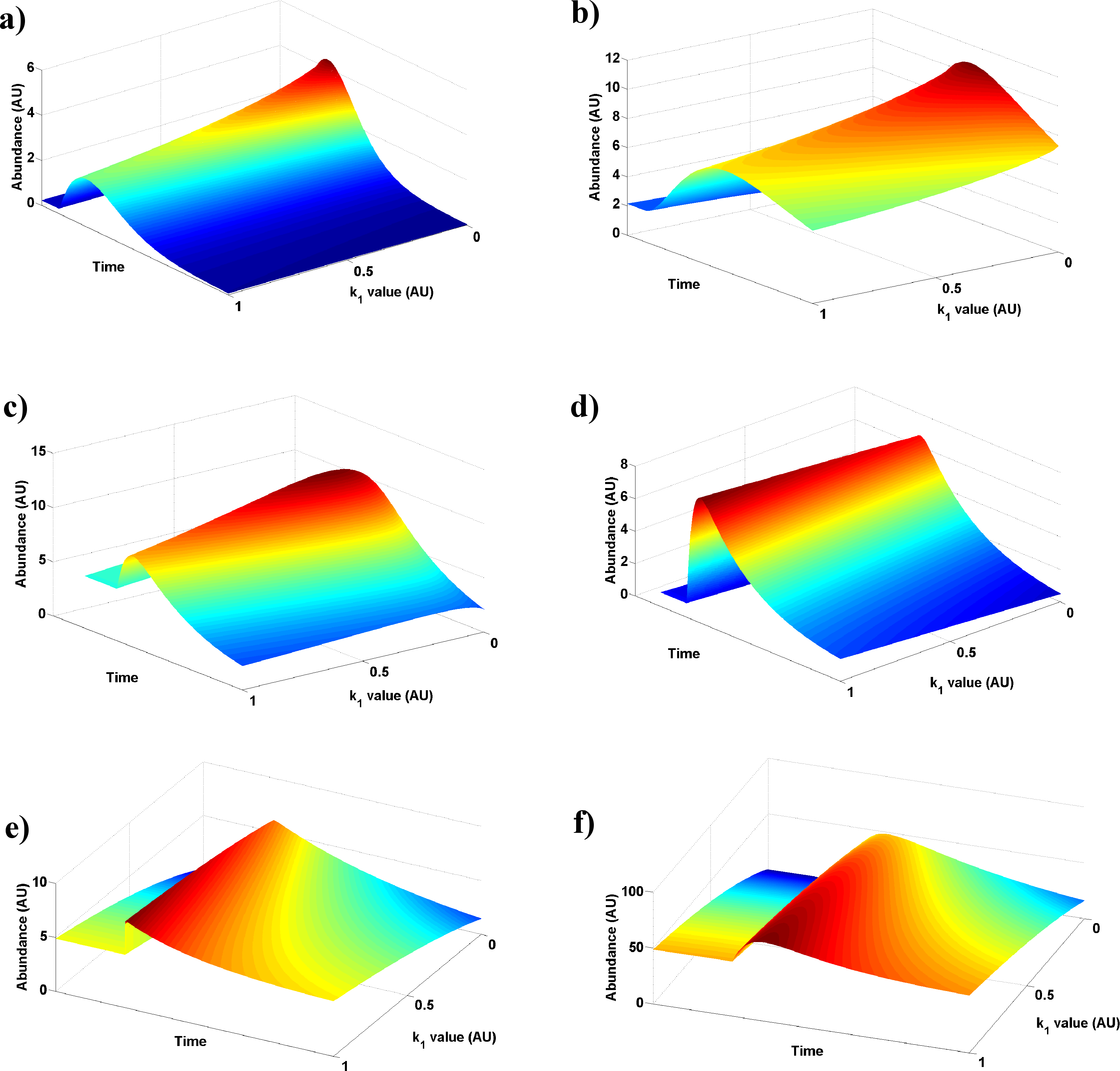
***

***Figure S2.*** *(Previous page) Response magnitude to an acute stimulus tails-off with increasing levels of oxidative stress in models containing the negative regulator (NegReg) species. Parameter scans were undertaken for rate constant k1 controlling oxidant generation flux.* ***a)*** *Time course simulation for ‘Function’ molecules in Model 2 under different values of k1.* ***b)*** *Time course simulation for ‘NegReg’ molecules in Model 2 under different values of k1.* ***c)*** *Time course simulation for ‘Function’ molecules in Model 3 under different values of k1.* ***d)*** *Time course simulation for ‘NegReg’ molecules in Model 3 under different values of k1.* ***e)*** *Time course simulation for ‘Activator’ molecules in Model 1 under different values of k1.* ***f)*** *Time course simulation for ‘Function’ molecules in Model 1 under different values of k1. Scan involves a regular step-wise increase in parameter value at 1000 intervals between 0 and 1. Stimulus strength =100.*

***
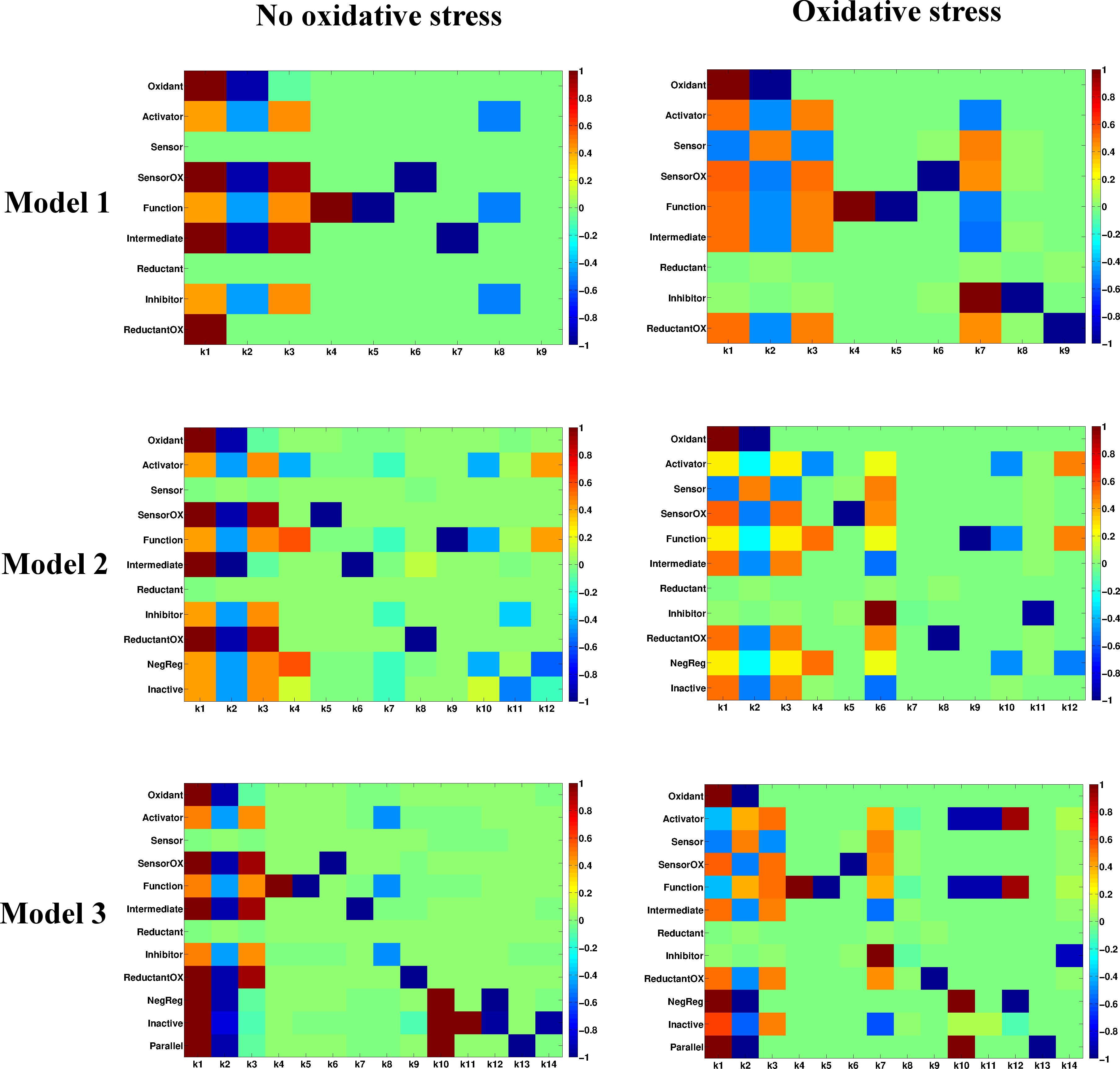
Figure S3.*** *Oxidative stress results in a shift in network sensitivities****.*** *Sensitivity analysis for Models 1-3 in the presence and absence of oxidative stress. Oxidative stress corresponds to a k1 value of 1.*

***
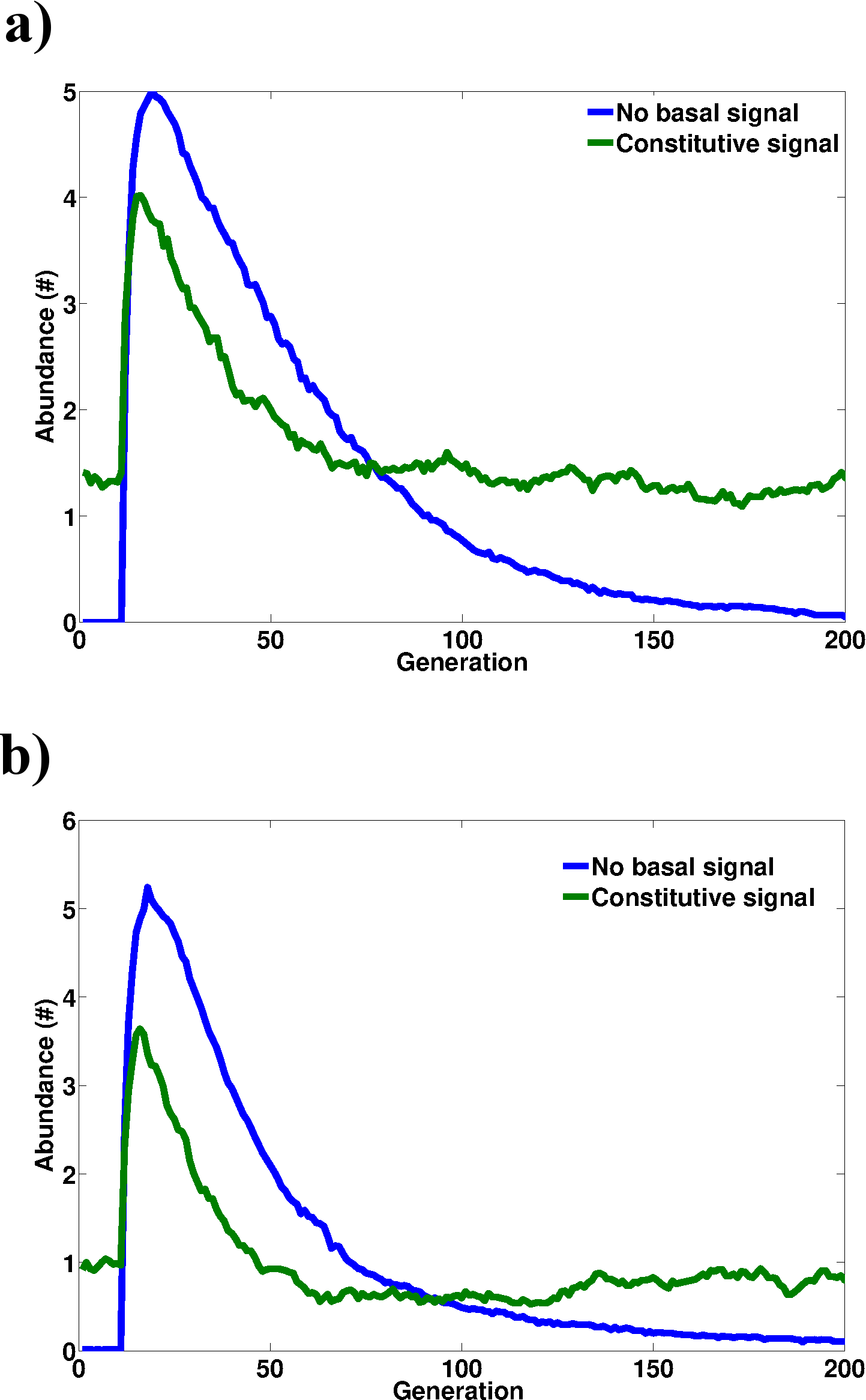
***

***Figure S4.*** *The dampening effect of constitutive signals in Models 2 and 3 is independent of the modelling framework used to simulate the models.* *A* *molecular dynamics simulation was performed for Model 2* ***(a)*** *and Model 3* ***(b)****. The average molecule abundance of ‘Activator’ molecules at each generation was derived from 1000 model runs. Initial molecule species were set to the same values as those specified in Supplementary Tables 6 and 9. All reactions were modelled to occur with a probability of 1 once the reactants collided in space. In the case of first order reactions, these were modelled to occur each generation. First order reactions include the resolving of the ‘intermediate’ molecule and the degradation of ‘NegReg’ in both models in addition to the degradation of ‘Function’ in Model 3. Such reactions occur with probabilities of 0.1, 0.01 and 0.05 respectively. Simulations were run at 50% spatial occupancy of the lattice space. Stimulus strength =100.*

***
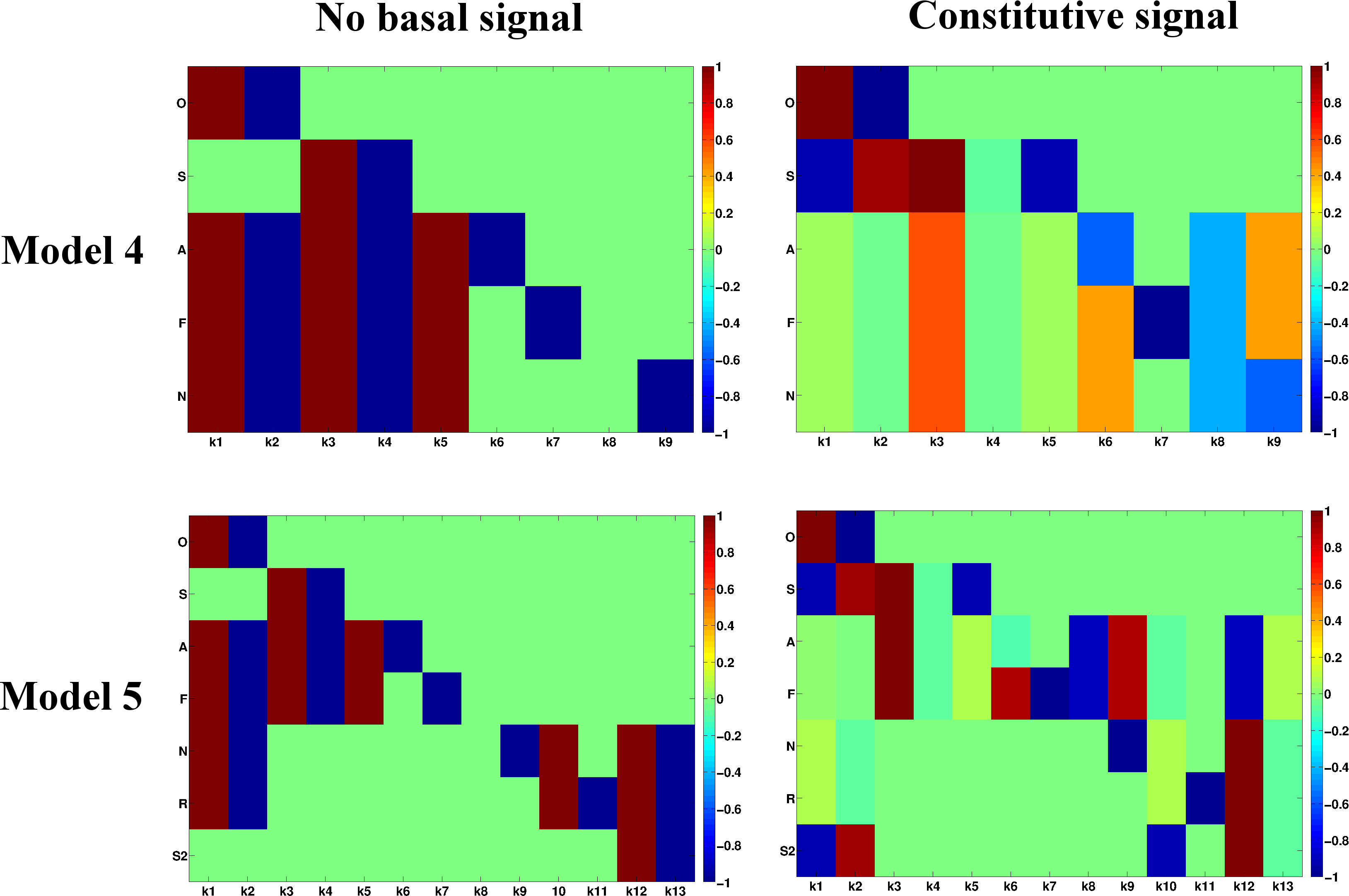
***

***Figure S5.*** *Constitutive signals* *result in a shift in network sensitivities in generic circuits of negative regulation. ‘O’,’S’,‘A’, ‘F’ and ‘N’ refer to molecular species in Models 4 and 5. Sensitivity analysis was performed for Models 4-5 in the presence and absence of a constitutive signal. A constitutive signal corresponds to a k1 value of 0.02.*

*
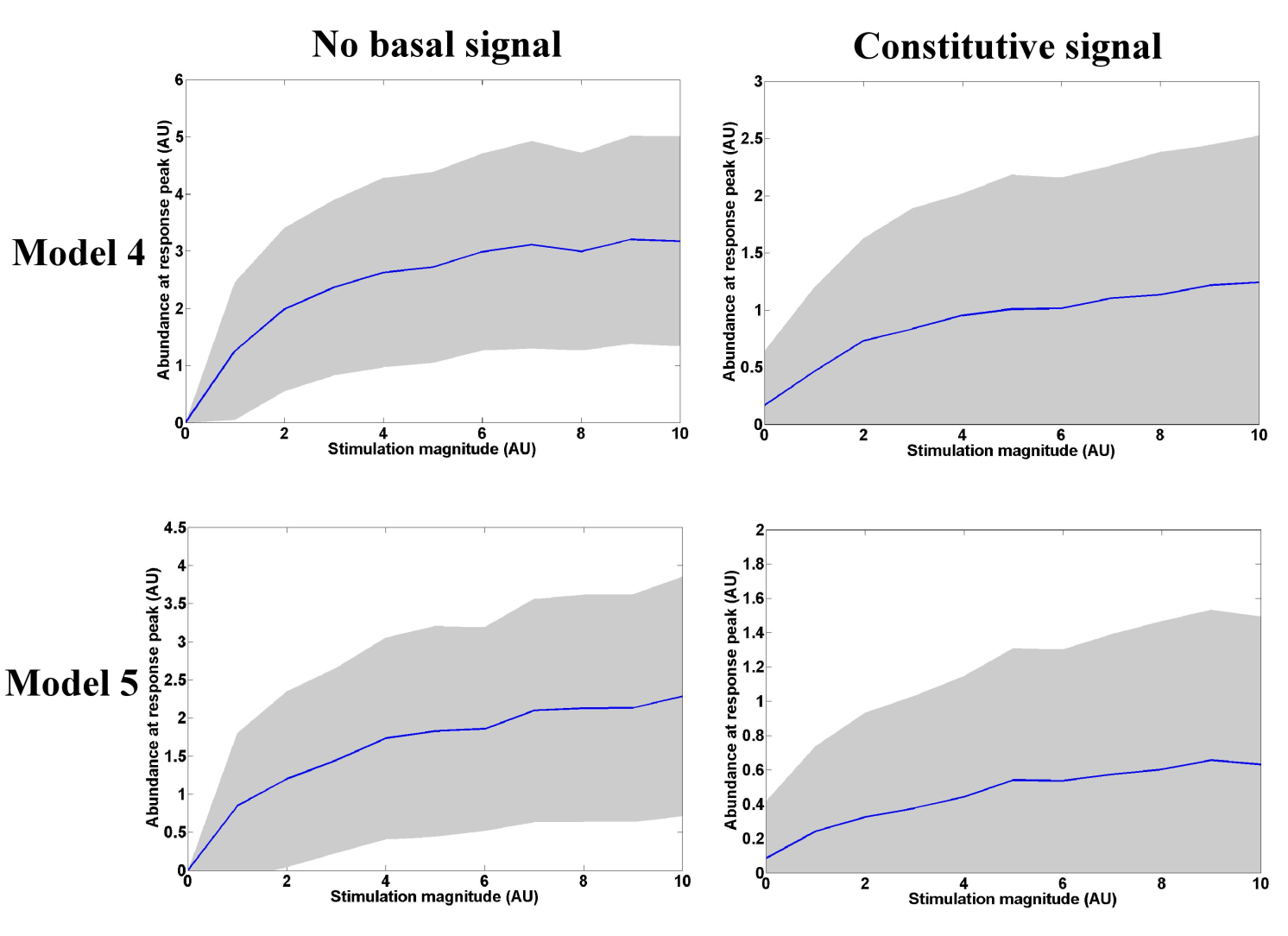
****Figure S6.*** *A sustained input increases response heterogeneity to an acute stimulus in generic circuits of negative regulation. Dose-response curves of molecule F in Models 4 and 5 were computed at different values of k1. Plots show the mean (blue) and standard deviation (shaded) of the abundance of molecule F at the peak of the pathway response derived from 1000 stochastic simulations. Constitutive signal corresponds to a k1 value of 0.02. k1 is the rate constant for O generation.*

## Supplementary Tables

| **Variable** | **Equation** |
| --- | --- |
| $d(\mathrm{Oxidant})/dt$ | $k_{1}-k_{2}\cdot AOX\cdot Oxidant-k_{3}\cdot Sensor\cdot Oxidant$ |
| $d(Sensor)/dt$ | $k_{8}\cdot Inhibitor\cdot Activator-k_{3}\cdot Sensor\cdot Oxidant$ |
| ${d(Sensor}_{OX})/dt$ | $k_{3}\cdot Sensor\cdot Oxidant-k_{6}\cdot{Sensor}_{OX}\cdot Reductant$ |
| $d(Activator)/dt$ | $k_{3}\cdot Sensor\cdot Oxidant-k_{8}\cdot Inhibitor\cdot Activator$ |
| $d(Function)/dt$ | $k_{4}\cdot Activator\cdot Relay-k_{5}\cdot Function$ |
| $d(Reductant)/dt$ | $k_{9}\cdot Reductant_{OX}-k_{6}\cdot{Sensor}_{OX}\cdot Reductant$ |
| $d(Intermediate)/dt$ | $k_{6}\cdot Sensor_{OX}\cdot Reductant-k_{7}\cdot Intermediate$ |
| $d(Inhibitor)/dt$ | $k_{7}\cdot Intermediate-k_{8}\cdot Inhibitor\cdot Activator$ |
| ${d(Reductant}_{OX})/dt$ | $k_{7}\cdot Intermediate-k_{9}\cdot Reductant_{OX}$ |

**Supplementary Table 1.** Ordinary differential equations in Model 1.

| **Rate Constant** | **Value** | **Reaction** |
| --- | --- | --- |
| $k_{1}$ | $0$ | Oxidant generation |
| $k_{2}$ | $0.1$ | Oxidant scavenging |
| $k_{3}$ | $0.1$ | Sensor oxidation |
| $k_{4}$ | $0.1$ | Relay reaction |
| $k_{5}$ | $0.1$ | Function decay |
| $k_{6}$ | $0.1$ | Sensor reduction |
| $k_{7}$ | $0.01$ | Resolving of intermediate |
| $k_{8}$ | $0.1$ | Inhibitory complex formation 1 |
| $k_{9}$ | $0.1$ | Reductant reduction |

**Supplementary Table 2.** Kinetic parameters in Model 1

**Supplementary Table 3.**  Species initial abundances in Model 1

| **Name** | **Initial abundance (AU)** |
| --- | --- |
| *Oxidant* | $0$ǂ |
| *Sensor* | $10$ |
| *Sensor_OX_* | $0$ |
| *Activator* | $0$ |
| *Function* | $0$ |
| *Reductant* | $100$ |
| *Intermediate* | $0$ |
| *Inhibitor* | $0$ |
| *Reductant_OX_* | $0$ |
| *AOX* | *100 (fixed)* |
| *Relay* | *10 (fixed)* |

ǂ Value raised to 100 during acute stimulus

| **Variable** | **Equation** |
| --- | --- |
| $d(\mathrm{Oxidant})/dt$ | $k_{1}-k_{2}\cdot AOX\cdot Oxidant-k_{3}\cdot Sensor\cdot Oxidant$ |
| $d(Sensor)/dt$ | $k_{7}\cdot Inhibitor\cdot Activator+k_{11}\cdot Inhibitor\cdot Inactive-k_{3}\cdot Sensor\cdot Oxidant$ |
| ${d(Sensor}_{OX})/dt$ | $k_{3}\cdot Sensor\cdot Oxidant-k_{5}\cdot{Sensor}_{OX}\cdot Reductant$ |
| $d(Activator)/dt$ | $k_{3}\cdot Sensor\cdot Oxidant-k_{7}\cdot Inhibitor\cdot Activator {- k}_{10}\cdot Activator\cdot NegReg$ |
| $d(Function)/dt$ | $k_{4}\cdot Activator\cdot Relay-k_{9}\cdot Function\cdot Relay2$ |
| $d(Reductant)/dt$ | $k_{8}\cdot Reductant_{OX}-k_{5}\cdot{Sensor}_{OX}\cdot Reductant$ |
| $d(Intermediate)/dt$ | $k_{5}\cdot Sensor_{OX}\cdot Reductant-k_{6}\cdot Intermediate$ |
| $d(Inhibitor)/dt$ | $k_{6}\cdot Intermediate-k_{7}\cdot Inhibitor\cdot Activator-k_{11}\cdot Inhibitor\cdot Inactive$ |
| ${d(Reductant}_{OX})/dt$ | $k_{6}\cdot Intermediate-k_{8}\cdot Reductant_{OX}$ |
| $d(NegReg)/dt$ | $k_{9}\cdot Function\cdot Relay2-k_{12}\cdot NegReg$ |
| $d(Inactive)/dt$ | $k_{10}\cdot Activator\cdot NegReg-k_{11}\cdot Inhibitor\cdot Inactive$ |

**Supplementary Table 4.** Ordinary differential equations in Model 2

**Supplementary Table 5.** Kinetic parameters in Model 2

| **Rate Constant** | **Value** | **Reaction** |
| --- | --- | --- |
| $k_{1}$ | $0$ | Oxidant generation |
| $k_{2}$ | $0.1$ | Oxidant scavenging |
| $k_{3}$ | $0.1$ | Sensor oxidation |
| $k_{4}$ | $0.1$ | Relay reaction |
| $k_{5}$ | $0.1$ | Sensor reduction |
| $k_{6}$ | $0.1$ | Resolving of intermediate |
| $k_{7}$ | $0.01$ | Inhibitory complex formation 1 |
| $k_{8}$ | $0.1$ | Reductant reduction |
| $k_{9}$ | $0.1$ | Relay reaction 2 |
| $k_{10}$ | $0.1$ | Inactivation of Activator |
| $k_{11}$ | $0.1$ | Inhibitory complex formation 2 |
| $k_{12}$ | $0.1$ | Degradation of negative regulator |

**Supplementary Table 6.**  Species initial abundances in Model 2.

| **Name** | **Initial abundance (AU)** |
| --- | --- |
| *Oxidant* | $0$ ǂ |
| *Sensor* | $10$ |
| *Sensor_OX_* | $0$ |
| *Activator* | $0$ |
| *Function* | $0$ |
| *Reductant* | $100$ |
| *Intermediate* | $0$ |
| *Inhibitor* | $0$ |
| *Reductant_OX_* | $0$ |
| *NegReg* | $0$ |
| *Inactive* | $0$ |
| *AOX* | *100 (fixed)* |
| *Relay* | *10 (fixed)* |
| *Relay2* | *10 (fixed)* |

ǂ Value raised to 100 during acute stimulus

| **Variable** | **Equation** |
| --- | --- |
| $d(\mathrm{Oxidant})/dt$ | $k_{1}-k_{2}\cdot AOX\cdot Oxidant-k_{3}\cdot Sensor\cdot Oxidant$ |
| $d(Sensor)/dt$ | $k_{8}\cdot Inhibitor\cdot Activator+k_{14}\cdot Inhibitor\cdot Inactive-k_{3}\cdot Sensor\cdot Oxidant$ |
| ${d(Sensor}_{OX})/dt$ | $k_{3}\cdot Sensor\cdot Oxidant-k_{6}\cdot{Sensor}_{OX}\cdot Reductant$ |
| $d(Activator)/dt$ | $k_{3}\cdot Sensor\cdot Oxidant-k_{8}\cdot Inhibitor\cdot Activator-k_{11}\cdot NegReg\cdot Activator$ |
| $d(Function)/dt$ | $k_{4}\cdot Activator\cdot Relay-k_{5}\cdot Function$ |
| $d(Reductant)/dt$ | $k_{9}\cdot Reductant_{OX}-k_{6}\cdot{Sensor}_{OX}\cdot Reductant$ |
| $d(Intermediate)/dt$ | $k_{6}\cdot Sensor_{OX}\cdot Reductant-k_{7}\cdot Intermediate$ |
| $d(Inhibitor)/dt$ | $k_{7}\cdot Intermediate-k_{8}\cdot Inhibitor\cdot Activator-k_{14}\cdot Inhibitor\cdot Inactive$ |
| ${d(Reductant}_{OX})/dt$ | $k_{7}\cdot Intermediate-k_{9}\cdot Reductant_{OX}$ |
| $d(Parallel)/dt$ | $k_{10}\cdot Oxidant\cdot Sensor2-k_{13}\cdot Parallel\cdot ParRelay$ |
| $d(NegReg)/dt$ | $k_{13}\cdot Parallel\cdot ParRelay-k_{12}\cdot NegReg$ |
| $d(Inactive)/dt$ | $k_{11}\cdot NegReg\cdot Activator-k_{14}\cdot Inhibitor\cdot Inactive$ |

**Supplementary Table 7.** Ordinary differential equations in Model 3

**Supplementary Table 8.** Kinetic parameters in Model 3

| **Rate Constant** | **Value** | **Reaction** |
| --- | --- | --- |
| $k_{1}$ | $0$ | Oxidant generation |
| $k_{2}$ | $0.1$ | Oxidant scavenging |
| $k_{3}$ | $0.1$ | Sensor oxidation |
| $k_{4}$ | $0.1$ | Relay reaction |
| $k_{5}$ | $0.1$ | Function decay |
| $k_{6}$ | $0.1$ | Sensor reduction |
| $k_{7}$ | $0.01$ | Resolving of intermediate |
| $k_{8}$ | $0.1$ | Inhibitory complex formation 1 |
| $k_{9}$ | $0.1$ | Reductant reduction |
| $k_{10}$ | $0.1$ | Sensor2 oxidation |
| $k_{11}$ | $0.1$ | Inactivation of Activator |
| $k_{12}$ | $0.1$ | Degradation of negative regulator |
| $k_{13}$ | $0.1$ | Parallel relay reaction |
| $k_{14}$ | $0.1$ | Inhibitory complex formation 2 |

| **Name** | **Initial abundance (AU)** |
| --- | --- |
| *Oxidant* | $0$ ǂ |
| *Sensor* | $10$ |
| *Sensor_OX_* | $0$ |
| *Activator* | $0$ |
| *Function* | $0$ |
| *Reductant* | $100$ |
| *Intermediate* | $0$ |
| *Inhibitor* | $0$ |
| *Reductant_OX_* | $0$ |
| *Parallel* | $0$ |
| *NegReg* | $0$ |
| *Inactive* | $0$ |
| *AOX* | *100 (fixed)* |
| *Relay* | *10 (fixed)* |
| *Sensor2* | *10 (fixed)* |
| *ParRelay* | *10 (fixed)* |

**Supplementary Table 9.**  Species initial abundances in Model 3.

ǂ Value raised to 100 during acute stimulus

**Supplementary Table 10.** Ordinary differential equations in Model 4

| **Variable** | **Equation** |
| --- | --- |
| $dO/dt$ | $k_{1}-k_{2}\cdot O$ |
| $dS/dt$ | $k_{3}-k_{4}\cdot S-k_{5}\cdot O\cdot S$ |
| $dA/dt$ | $k_{5}\cdot O\cdot S-k_{6}\cdot A-k_{8}\cdot A\cdot N$ |
| $dF/dt$ | $k_{6}\cdot A-k_{7}\cdot F$ |
| $dN/dt$ | $k_{7}\cdot F-k_{9}\cdot N$ |

**Supplementary Table 11.** Kinetic parameters in Model 4

| **Rate Constant** | **Value** |
| --- | --- |
| $k_{1}$ | $0$ |
| $k_{2}$ | $0.1$ |
| $k_{3}$ | $0.1$ |
| $k_{4}$ | $0.01$ |
| $k_{5}$ | $0.1$ |
| $k_{6}$ | $0.1$ |
| $k_{7}$ | $0.01$ |
| $k_{8}$ | $0.1$ |
| $k_{9}$ | $0.01$ |

**Supplementary Table 12.**  Species initial abundances in Model 4

| **Name** | **Initial abundance (AU)** |
| --- | --- |
| *O* | $0$ ǂ |
| *S* | $10$ |
| *A* | $0$ |
| *F* | $0$ |
| *N* | $0$ |

ǂ Value raised to 100 during acute stimulus

**Supplementary Table 13.** Ordinary differential equations in Model 5

| **Variable** | **Equation** |
| --- | --- |
| $dO/dt$ | $k_{1}-k_{2}\cdot O$ |
| $dS/dt$ | $k_{3}-k_{4}\cdot S-k_{5}\cdot O\cdot S$ |
| $dA/dt$ | $k_{5}\cdot O\cdot S-k_{6}\cdot A-k_{8}\cdot A\cdot N$ |
| $dF/dt$ | $k_{6}\cdot A-k_{7}\cdot F$ |
| $dS2/dt$ | $k_{12}-k_{13}\cdot S2-k_{10}\cdot O\cdot S2$ |
| $dR/dt$ | $k_{10}\cdot O\cdot S2-k_{11}\cdot R$ |
| $dN/dt$ | $k_{11}\cdot R-k_{9}\cdot N$ |

**Supplementary Table 14.** Kinetic parameters in Model 5

| **Rate Constant** | **Value** |
| --- | --- |
| $k_{1}$ | $0$ |
| $k_{2}$ | $0.1$ |
| $k_{3}$ | $0.1$ |
| $k_{4}$ | $0.01$ |
| $k_{5}$ | $0.1$ |
| $k_{6}$ | $0.1$ |
| $k_{7}$ | $0.1$ |
| $k_{8}$ | $0.1$ |
| $k_{9}$ | $0.01$ |
| $k_{10}$ | 0 |
| $k_{11}$ | 0 |
| $k_{12}$ | 0 |
| $k_{13}$ | 0.01 |

**Supplementary Table 15.**  Species initial abundances in Model 5

| **Name** | **Initial abundance (AU)** |
| --- | --- |
| *O* | $0$ ǂ |
| *S* | $10$ |
| *A* | $0$ |
| *F* | $0$ |
| *S2* | $10$ |
| *R* | $0$ |
| *N* | $0$ |

ǂ Value raised to 100 during acute stimulus
